# Validity of a Self-administered Food Frequency Questionnaire Used in the 5-year Follow-up Survey of the JPHC Study Cohort I: Comparison with Dietary Records for Food Groups

**DOI:** 10.2188/jea.13.1sup_57

**Published:** 2007-11-30

**Authors:** Satoshi Sasaki, Minatsu Kobayashi, Shoichiro Tsugane

**Affiliations:** Epidemiology and Biostatistics Division, National Cancer Center Research Institute East.

**Keywords:** Validity, food group, food frequency questionnaire, dietaryrecord

## Abstract

We examined the validity of food intake measurements from a self-administered food frequency questionnaire (FFQ) used in the 5-year follow-up survey of the JPHC study using 28- or 14-day dietary records (DR) as the gold standard. The median (range) correlation coefficients between a 19-food group measured by FFQ and DR were 0.42 (0.13-0.76) for men and 0.41 (0.02-0.75) for women. The median (range) for energy-adjusted correlation coefficients was 0.38 (0.08-0.76) for men and 0.32 (0.06-0.66) for women. The mean percentage of classification into the same categories between the two methods was 30% in men and 31% in women. Only 2% in men and 3% in women of subjects were classified into the extreme opposite categories. When we adjusted for area, the median correlation coefficients were decreased in crude intakes (0.34 in men and 0.28 in women), whereas no remarkable change was observed in energy-adjusted intakes (0.33 in men and 0.29 in women). In conclusion, the results suggest that the FFQ can be used in the JPHC study cohort I to rank individuals according to the intakes for most of the food groups examined. But intake levels both at population and individual levels were difficult to estimate.

Food intakes have been assessed with a self-administered semiquantitative food frequency questionnaire (FFQ) in several nutritional epidemiologic studies for chronic diseases. But the validity issue has received little attention in studies performed for various Japanese populations. Although recently developed questionnaires for nutritional epidemiology have usually been designed to compute several types of nutrients rather than foods and food groups, the validity of intake by food groups of a newly developed questionnaire is as interesting and important as that of nutrient intakes because foods and food groups are used as factors in several epidemiologic studies as well as nutrients, and the results for foods and food groups can be used directly for public health policies. Notwithstanding this importance, the validity of food frequency questionnaires for foods and food groups has not been reported in as many studies compared to that for nutrients.^1^^-^^7^

In here, we examined the validity of food group intakes assessed with FFQ using 28- or 14-day dietary records (DR).

## METHODS

The study design and subject characteristics have been reported elsewhere.^8^ The subjects included in the analysis were 102 men and 113 women who completed 28-day DRs in Iwate, Akita, and Nagano, and 14-day DRs in Okinawa, and then answered the FFQ. The DR survey method and the method for computing food intakes from FFQ have been described elsewhere.^9^^-^^10^ We compared the mean intakes and computed Spearman rank correlation coefficients for 19 food groups. Vegetables were further divided into two groups, green and yellow vegetables and pickled vegetables. Beverages were reported in two groups, alcoholic and non-alcoholic. The intake of sugars and sweeteners was not computed in the FFQ, so this food group was not included in the present analysis.

For the computation of intakes from DR, means of 28- or 14-day intakes were used as representative values in this study. Crude and energy-adjusted values were used for computation of the correlation coefficients. A residual model was used for energy-adjustment.^11^

Spearman rank correlation coefficient was used for the correlation analysis because the distribution was skewed in most food groups. In order to validate categorization of the subjects into quintiles by values obtained from the FFQ, we computed the mean intakes from DRs by categories determined by nutrient intakes from the FFQ. Moreover, in order to examine the validity of categorization in another way, we computed the number of subjects classified into same, adjacent, and extreme categories by joint classification by quintiles. Since the aim was to quantify measurement error rather than test a hypothesis, p values were not presented. All the analyses were performed on men and women separately. The computation was performed using the data on 4 areas combined. We additionally computed partial correlation coefficients, adjusting for using dummy variables.

## RESULTS

Table 1 shows the intakes of food groups assessed with two methods, and their correlation coefficients. For mean intakes, the percent difference varied from -41% for confectioneries to +69% for fruits in men and -45% for confectioneries to +78% for algae in women except for non-alcohólic beverages, and seasonings and spices. Those two foods were excluded from the comparison because assessment methods (i.e., the rules of food weighing and food coding) were different for each of the two methods. The correlation coefficients in crude values varied from 0.13 for fats and oils to 0.76 for alcoholic beverages in men and 0.02 for algae to 0.75 for pickled vegetables in women. The median was 0.42 in men and 0.41 in women. The correlation coefficients in energy-adjusted values varied from 0.08 for algae to 0.76 for alcoholic beverages in men and 0.06 for algae and 0.64 for milks in women. The median was 0.38 in men and 0.32 in women. When the area was adjusted for, the median correlation coefficients were similar between crude and energy-adjusted intakes: 0.34 and 0.33, respectively, in men, and 0.28 and 0.29, respectively, in women.

**Table 1.** Food group intakes (g/day) assessed with DR for 28- or 14-days and FFQ in 4 areas and their correlations

Sex Food group	DR				FFQ				%		Spearman correlation coefficient		
		
	Mean	±	SD	Median	Mean	±	SD	Median	difference^1^	Crude	Energy-djusted^2^	Area-adjusted^3^	Energy- and area-adjusted^2,3^
Men (n=102)													
Cereals	313	±	86	296	349	±	118	340	11	0.59	0.42	0.55	0.48
Potatoes and starches	51	±	27	47	32	±	38	22	-37	0.22	0.33	0.22	0.31
Confectioneries	28	±	26	21	17	±	22	11	-41	0.51	0.48	0.45	0.43
Fats and oils	11	±	4	10	14	±	7	13	23	0.13	0.24	0.11	0.07
Nuts and seeds	2	±	3	1	2	±	4	1	-1	0.45	0.26	0.29	0.28
Pulses	87	±	36	82	78	±	51	67	-11	0.42	0.53	0.36	0.45
Fish and shellfish	136	±	46	137	114	±	85	83	-16	0.46	0.32	0.32	0.30
Meats	76	±	32	69	71	±	48	57	-7	0.42	0.50	0.40	0.37
Eggs	39	±	14	38	33	±	20	25	-16	0.28	0.25	0.23	0.23
Milk and dairy products	124	±	110	109	194	±	237	144	57	0.66	0.52	0.63	0.56
Vegetables	314	±	89	307	250	±	189	215	-21	0.33	0.22	0.33	0.26
Green and yellow	108	±	48	100	98	±	93	77	-10	0.38	0.38	0.34	0.32
Pickled^4^	34	±	30	25	49	±	64	23	44	0.74	0.54	0.37	0.33
Fruits	120	±	85	95	204	±	231	141	69	0.61	0.41	0.51	0.43
Fungi	9	±	6	7	10	±	9	8	20	0.40	0.44	0.33	0.43
Algae	8	±	6	6	12	±	12	9	53	0.22	0.08	0.22	0.11
Alcoholic beverages	309	±	276	260	308	±	339	180	-1	0.76	0.76	0.76	0.77
Non-alcoholic beverages	272	±	134	263	906	±	611	713	233	0.44	0.32	0.42	0.34
Seasonings and spices	36	±	10	35	4	±	4	3	-88	0.25	0.12	0.07	0.11
Median	---		---			---		---	---	0.42	0.38	0.34	0.33
Women(n=113)													
Cereals	220	±	49	217	294	±	95	276	33	0.43	0.27	0.37	0.27
Potatoes and starches	49	±	23	44	38	±	43	28	-22	0.09	0.20	0.06	0.17
Confectioneries	49	±	32	46	27	±	36	16	-45	0.52	0.38	0.45	0.31
Fats and oils	10	±	4	9	14	±	10	11	48	0.06	0.21	0.06	0.04
Nuts and seeds	3	±	5	1	3	±	7	1	-2	0.41	0.15	0.20	0.20
Pulses	74	±	29	71	74	±	45	61	0	0.42	0.49	0.31	0.44
Fish and shellfish	106	±	33	102	105	±	103	80	-1	0.42	0.32	0.24	0.29
Meats	60	±	29	52	58	±	41	47	-3	0.29	0.45	0.36	0.41
Eggs	33	±	12	32	31	±	19	25	-7	0.43	0.42	0.43	0.43
Milk and dairy products	152	±	94	155	207	±	180	200	37	0.68	0.64	0.67	0.65
Vegetables	300	±	91	289	284	±	238	233	-6	0.35	0.32	0.31	0.34
Green and yellow	111	±	48	103	115	±	95	89	3	0.37	0.32	0.28	0.25
Pickled^4^	30	±	26	22	48	±	61	27	58	0.75	0.57	0.42	0.34
Fruits	156	±	84	144	264	±	299	178	69	0.50	0.23	0.38	0.25
Fungi	8	±	6	7	12	±	10	11	53	0.31	0.38	0.23	0.35
Algae	7	±	5	5	13	±	9	11	78	0.02	0.06	0.03	0.09
Alcoholic beverages	24	±	41	8	20	±	70	0	-18	0.52	0.50	0.49	0.51
Non-alcoholic beverages	279	±	114	270	799	±	506	686	186	0.30	0.32	0.28	0.30
Seasonings and spices	33	±	10	33	5	±	5	4	-85	0.20	0.12	0.04	0.19
Median		---		---		---		---	---	0.41	0.32	0.28	0.29

^1^(FFQ - DR)/DR (%). ^2^Energy was adjusted by residual method. ^3^Area was adjusted using dummy variables.

^4^Pickled plum (umeboshi) was included in pickled vegetables, and not in total vegetables but rather in fruits.

For n=102, r>0.20 = p<0.05, r>0.26 = p<0.01, r>0.33 = p<0.001. For n=113, r>0.19 = p<0.05, r>0.25 = p<0.01, r>0.31 = p<0.001.

Table 2 shows food group intakes assessed with DR within quintile of intake assessed with the FFQ. The mean intake in the highest quintile was five times higher or more than in the lowest quintile for nuts and seeds, and pickled vegetables in both sexes, and for alcoholic beverages and dairy products in men. A steady increase in mean intake from the lowest to the highest quintile was observed for cereals, fish and shellfish, meats, milk and dairy products, vegetables, pickled vegetables and alcoholic beverage in men, and for confectioneries, pulses, milk and dairy products and pickled vegetables in women.

**Table 2.** Food group intakes from DR within quintile of intake determined by FFQ

Sex	Quintile of food groups intake according to FFQ																							

	Lowest				2nd					3rd					4th					Highest				
					
Food group	(n^1^)	Mean	±	SD	(n)	Mean	±	SD	Ratio^2^	(n)	Mean	±	SD	Ratio^2^	(n)	Mean	±	SD	Ratio^2^	(n)	Mean	±	SD	Ratio^2^
Men (n=102)																								
Cereals	(20)	242	±	43	(21)	289	±	77	1.20	(20)	318	±	63	1.32	(21)	331	±	67	1.37	(20)	385	±	105	1.59
Potatoes and starches	(20)	49	±	30	(21)	45	±	21	0.92	(20)	48	±	13	0.99	(21)	46	±	15	0.94	(20)	68	±	41	1.41
Confectioneries	(21)	15	±	18	(21)	17	±	12	1.15	(19)	17	±	15	1.15	(21)	39	±	30	2.58	(20)	52	±	30	3.44
Fats and oils	(20)	10	±	4	(21)	11	±	5	1.12	(20)	12	±	5	1.26	(21)	13	±	3	1.30	(20)	11	±	5	1.09
Nuts and seeds	(32)	1	±	1	(14)	2	±	2	2.69	(23)	2	±	2	3.59	(11)	3	±	4	5.08	(22)	4	±	6	6.65
Pulses	(20)	65	±	32	(21)	72	±	26	1.11	(20)	91	±	25	1.40	(21)	104	±	43	1.60	(20)	104	±	37	1.60
Fish and shellfish	(20)	101	±	36	(21)	127	±	41	1.26	(20)	142	±	42	1.41	(21)	137	±	37	1.35	(20)	170	±	48	1.68
Meats	(20)	54	±	23	(21)	71	±	29	1.31	(20)	75	±	24	1.38	(21)	82	±	33	1.52	(20)	100	±	35	1.86
Eggs	(21)	36	±	14	(34)	36	±	12	0.99	(13)	41	±	9	1.14	(34)	45	±	15	1.25	---		---		---
Milk and dairy products	(20)	42	±	46	(21)	76	±	72	1.80	(22)	104	±	73	2.46	(19)	174	±	87	4.11	(20)	229	±	139	5.44
Vegetables	(21)	284	±	82	(21)	297	±	79	1.05	(19)	299	±	92	1.05	(21)	320	±	95	1.13	(20)	372	±	77	1.31
Green and yellow	(21)	84	±	41	(21)	108	±	38	1.29	(19)	102	±	43	1.22	(21)	113	±	54	1.36	(20)	135	±	50	1.61
Pickled^3^	(21)	11	±	12	(21)	14	±	13	1.23	(19)	28	±	22	2.53	(21)	53	±	24	4.80	(20)	65	±	29	5.86
Fruits	(21)	77	±	62	(21)	74	±	36	0.96	(19)	108	±	71	1.40	(21)	124	±	55	1.60	(20)	219	±	99	2.83
Fungi	(23)	6	±	4	(16)	5	±	3	0.85	(23)	9	±	7	1.42	(18)	12	±	7	1.98	(22)	11	±	6	1.91
Algae	(21)	5	±	4	(20)	8	±	5	1.47	(24)	7	±	5	1.29	(17)	10	±	7	1.83	(20)	9	±	8	1.66
Alcoholic beverages	(23)	73	±	126	(18)	163	±	154	2.22	(19)	326	±	171	4.44	(22)	358	±	174	4.88	(20)	644	±	307	8.78
Non-alcoholic beverages	(20)	191	±	79	(21)	239	±	125	1.25	(20)	236	±	106	1.24	(21)	325	±	158	1.70	(20)	367	±	118	1.92
Seasonings and spices	(19)	33	±	12	(22)	36	±	10	1.10	(20)	33	±	10	1.02	(21)	38	±	11	1.16	(20)	39	±	10	1.19
Women (n=113)																								
Cereals	(22)	189	±	35	(23)	211	±	39	1.12	(23)	208	±	36	1.10	(23)	237	±	40	1.25	(22)	258	±	62	1.37
Potatoes and starches	(22)	52	±	23	(23)	44	±	20	0.83	(23)	38	±	19	0.73	(23)	55	±	22	1.05	(22)	54	±	27	1.04
Confectioneries	(23)	29	±	25	(22)	37	±	26	1.29	(23)	49	±	30	1.72	(23)	56	±	19	1.98	(22)	75	±	39	2.62
Fats and oils	(22)	9	±	4	(23)	10	±	3	1.12	(23)	10	±	3	1.09	(23)	10	±	3	1.06	(22)	10	±	4	1.11
Nuts and seeds	(27)	1	±	1	(28)	2	±	2	3.38	(24)	2	±	3	3.37	(13)	6	±	11	8.56	(21)	4	±	3	5.38
Pulses	(22)	51	±	22	(23)	72	±	23	1.41	(23)	76	±	22	1.47	(23)	78	±	29	1.52	(22)	94	±	31	1.82
Fish and shellfish	(22)	84	±	29	(23)	93	±	30	1.11	(23)	117	±	28	1.40	(23)	116	±	30	1.39	(22)	120	±	34	1.43
Meats	(22)	47	±	23	(23)	53	±	22	1.12	(23)	64	±	39	1.36	(23)	70	±	28	1.48	(22)	64	±	27	1.35
Eggs	(29)	26	±	10	(6)	35	±	9	1.37	(29)	31	±	9	1.22	(16)	36	±	12	1.40	(33)	40	±	14	1.55
Milk and dairy products	(22)	66	±	68	(23)	110	±	49	1.66	(23)	152	±	78	2.29	(23)	189	±	62	2.85	(22)	241	±	98	3.64
Vegetables	(22)	258	±	64	(23)	273	±	71	1.06	(23)	323	±	125	1.25	(23)	302	±	72	1.17	(22)	347	±	85	1.34
Green and yellow	(22)	86	±	32	(23)	107	±	38	1.23	(23)	103	±	49	1.19	(23)	114	±	45	1.32	(22)	147	±	57	1.70
Pickled^3^	(22)	8	±	9	(23)	15	±	12	2.00	(23)	27	±	19	3.54	(23)	46	±	19	6.02	(22)	56	±	29	7.33
Fruits	(22)	84	±	52	(23)	163	±	82	1.95	(23)	143	±	68	1.71	(23)	180	±	62	2.15	(22)	211	±	99	2.52
Fungi	(22)	5	±	4	(22)	7	±	5	1.44	(22)	9	±	5	1.87	(29)	9	±	5	1.91	(18)	10	±	7	2.00
Algae	(22)	7	±	6	(23)	8	±	5	1.19	(23)	5	±	4	0.79	(23)	9	±	7	1.27	(22)	6	±	4	0.94
Alcoholic beverages	---		---		---		---		---	---		---		---	---		---		---	---		---		---
Non-alcoholic beverages	(22)	208	±	95	(23)	292	±	108	1.41	(23)	278	±	111	1.34	(23)	294	±	110	1.42	(22)	323	±	121	1.56
Seasonings and spices	(22)	29	±	10	(23)	33	±	11	1.16	(24)	32	±	10	1.10	(22)	35	±	10	1.22	(22)	35	±	9	1.21

^1^Number of each subject.

^2^Ratio compared to the lowest quintile.

^3^Pickled plum (umeboshi) was included in pickled vegetables, and not in total vegetables but rather in fruits.

Table 3 shows the comparison of the FFQ with the DR based on joint classification by quintile. The mean percentage of classification into the same categories between the two methods was 30% in men and 31% in women. Only 2% in male and 3% in female subjects were classified into the extreme opposite categories.

**Table 3.** Comparison of FFQ with DR for food group intakes based on joint classification by quintile (%).

Food group	Men (n=102)			Women (n=113)		
	Same category	Adjacent category	Extreme category	Same category	Adjacent category	Extreme category
Cereals	25	52	1	37	32	2
Potatoes and starches	24	37	3	14	39	7
Confectioneries	37	34	2	36	35	3
Fats and oils	29	30	8	24	31	8
Nuts and seeds	33	32	1	30	33	1
Pulses	31	39	3	36	30	2
Fish and shellfish	30	36	1	36	31	3
Meats	31	39	1	29	37	3
Eggs	22	41	4	29	41	4
Milk and dairy products	37	45	1	35	44	1
Vegetables	29	31	4	24	38	2
Green and yellow	28	33	3	33	35	2
Pickled^1^	40	42	0	44	42	0
Fruits	37	40	1	35	35	2
Fungi	29	38	3	24	42	3
Algae	28	24	4	21	30	6
Alcoholic beverages	53	31	1	---	---	---
Non-alcoholic beverages	32	37	0	31	30	4
Seasonings and spices	24	35	5	22	34	4
Median	30	37	2	31	36	3

^1^Pickled plum (umeboshi) was included in pickled vegetables, and not in total vegetables but rather in fruits.

Table 4 shows Spearman rank correlation coefficients for food groups when assessed with DR and FFQ. For crude value, the ranges were from 0.13 for fats and oils to 0.76 for alcoholic beverages in men, and from 0.02 for algae to 0.75 for pickled vegetables in women. For energy-adjusted value, the ranges were from 0.08 for algae to 0.76 for alcoholic beverages in men, and from 0.06 for algae to 0.66 for milks in women. The directions of the change after energy adjustment were different among food groups.

**Table 4.** Spearman rank correlation coefficients for food groups between values assessed with DR and FFQ

	Crude					Energy-adjusted^1^				
	
	Iwate	Akita	Nagano	Okinawa	Total	Iwate	Akita	Nagano	Okinawa	Total
Men	(n=24)	(n=28)	(n=23)	(n=27)	(n=102)	(n=24)	(n=28)	(n=23)	(n=27)	(n=102)
Cereals	0.34	0.64	0.71	0.16	0.64	0.48	0.46	0.71	0.15	0.45
Potatoes and starches	0.38	0.04	0.43	0.06	0.22	0.45	0.10	0.65	0.16	0.33
Confectiorienes	0.51	0.75	0.47	0.09	0.51	0.39	0.56	0.49	0.11	0.48
Fats and oils	0.05	0.05	0.06	0.08	0.13	-0.09	0.04	0.05	0.16	0.24
Nuts and seeds	0.13	0.33	0.53	0.19	0.45	0.10	0.45	0.35	0.29	0.26
Pulses	0.63	-0.01	0.41	0.32	0.45	0.55	0.32	0.35	0.32	0.54
Fish and shellfish	0.50	0.31	0.26	0.20	0.45	0.33	0.51	0.12	0.11	0.32
Meats	0.52	0.46	0.21	0.52	0.42	0.39	0.54	0.27	0.48	0.50
Eggs	0.40	0.53	-0.21	0.23	0.28	0.31	0.45	0.24	0.17	0.25
Milk and dairy products	0.69	0.77	0.63	0.51	0.66	0.58	0.62	0.61	0.31	0.52
Vegetables	0.34	0.23	0.31	0.51	0.36	0.21	0.33	0.42	0.30	0.23
Green & yellow	0.43	0.30	0.07	0.45	0.38	0.31	0.43	0.34	0.18	0.36
Pickled^2^	0.44	0.36	0.46	-0.13	0.74	0.49	0.19	0.33	-0.19	0.54
Fruits	0.64	0.73	-0.13	0.35	0.61	0.61	0.68	-0.04	0.21	0.41
Fungi	0.23	0.29	0.38	0.25	0.40	0.36	0.37	0.49	0.35	0.44
Algae	0.21	0.31	0.35	0.19	0.22	0.12	0.19	0.10	0.12	0.08
Alcoholic beverages	0.89	0.57	0.81	0.81	0.76	0.79	0.63	0.81	0.75	0.76
Non-alcoholic beverages	0.50	0.50	0.35	0.16	0.43	0.37	0.42	0.26	0.07	0.33
Seasonings and spices	0.29	-0.13	0.31	0.14	0.28	0.44	-0.08	0.17	-0.03	0.09
Median	0.43	0.33	0.35	0.20	0.43	0.39	0.43	0.34	0.17	0.36
Women	(n=27)	(n=30)	(n=28)	(n=28)	(n=113)	(n=27)	(n=30)	(n=28)	(n=28)	(n=113)
Cereals	0.36	0.28	0.59	0.24	0.44	0.24	0.20	0.59	0.01	0.27
Potatoes and starches	0.22	-0.02	0.16	-0.19	0.09	0.24	0.04	0.36	-0.14	0.20
Confectioneries	0.30	0.53	0.55	0.45	0.52	0.33	0.40	0.36	0.17	0.39
Fats and oils	0.23	0.02	0.16	0.03	0.06	-0.19	0.30	0.01	0.35	0.21
Nuts and seeds	0.12	0.31	0.41	-0.04	0.41	-0.04	0.22	0.36	0.04	0.15
Pulses	0.38	0.25	0.39	0.30	0.40	0.58	0.42	0.14	0.27	0.49
Fish and shellfish	0.51	-0.03	0.29	0.15	0.42	0.41	0.15	0.22	0.18	0.32
Meats	0.52	0.55	0.22	0.26	0.29	0.35	0.51	0.36	0.29	0.45
Eggs	0.46	0.69	0.48	0.07	0.43	0.50	0.58	0.48	0.21	0.42
Milk and dairy products	0.66	0.75	0.78	0.55	0.69	0.43	0.66	0.79	0.57	0.66
Vegetables	0.53	0.07	0.24	0.38	0.34	0.37	0.44	0.32	0.38	0.31
Green & yellow	0.68	-0.06	0.20	0.15	0.32	0.51	0.22	0.16	0.07	0.30
Pickled^2^	0.53	0.27	0.63	0.22	0.75	0.47	0.25	0.62	0.11	0.57
Fruits	0.46	0.14	0.52	0.55	0.50	0.16	0.40	0.35	0.25	0.23
Fungi	0.02	0.28	0.19	0.54	0.31	0.0l	0.41	0.38	0.47	0.38
Algae	-0.08	-0.02	-0.01	0.07	0.02	-0.03	0.20	-0.08	0.04	0.06
Alcoholic beverages	0.75	0.59	0.47	0.23	0.52	0.58	0.11	0.43	0.08	0.50
Non-alcoholic beverages	0.39	0.24	0.27	0.24	0.28	0.58	0.13	0.27	0.23	0.30
Seasonings and spices	0.17	-0.27	0.07	0.30	0.21	0.20	-0.10	0.30	0.35	0.14
Median	0.39	0.25	0.29	0.24	0.40	0.35	0.25	0.36	0.21	0.31

^1^Energy was adjusted by residual method. ^2^Pickled plum was included.

## DISCUSSION

Validation studies of a newly developed dietary assessment questionnaire for food groups have been reported less often than for nutrients. The validation studies for two food frequency questionnaires developed in Europe reported that the median correlation coefficients between intakes assessed with the tested questionnaire and those assessed with twelve 24-hour dietary recalls were 0.61 in men and 0.59 in women in Spain, and 0.61 in men and 0.53 in women in the Netherlands.^3^^,^^5^ The median correlation coefficient in the FFQ developed in Japan with DR was 0.39^6^ and 0.46.^7^ The correlation coefficients observed in this study (median value was 0.42 in men and 0.41 in women) were slightly lower than the values observed in Europe and comparable with those in Japan. However, the correlation coefficients decreased in most of the food groups expressed as crude intakes when area was adjusted for using dummy variables. In contrast, the area-adjustment did not make for remarkable differences in correlation coefficients in energy-adjusted intakes. A possible area-based difference of response to dietary surveys, probably both to DR and FFQ, might have affected the examination of the validity of the FFQ.

Among food groups examined, cereals and dairy products, pickled vegetables, fruits, and alcoholic beverages showed relatively high correlations in both sexes (>=0.50 except for cereals in women) (Table 1). The high validity of this type of questionnaire for the assessment of these food groups has consistently been reported in previous studies.^4^^,^^5^ On the other hand, some studies reported a relatively low validity for fish,^4^^,^^5^ whereas the correlation coefficients for fish observed in this study were 0.46 in men and 0.42 in women, i.e., higher than the median values (Table 1). Higher intake of fish and the wider between-person variation in this study compared to the previous studies in Europe might have affected the observed higher level of validity in this study.

Several studies have indicated over-reporting by a questionnaire for socially desirable foods such as milk, especially low-fat milk,^4^^,^^5^ but other studies did not confirm this.^1^^,^^3^ In the present study, over-reporting was marked for dairy products and fruits, and under-reporting was pronounced for confectioneries. The results indicated that social desirability possibly biased the dietary intakes from the FFQ for these food groups.^12^ This should be considered when intakes are computed for the nutrients which are abundant in these food groups. One possible method to reduce this problem is to compute the social desirability bias (for example, as the ratio of true intake assessed with DR to the reported intake assessed with the FFQ for foods), and apply it to the estimation of the intake. However, because the bias may depend on subjects' characteristics, this method might be practically difficult.

In conclusion, although the observed correlation coefficients for food group intakes assessed with FFQ and 28- or 14-day DRs were slightly lower than the reported values in Western populations, the results were comparable with those reported in studies conducted in Japan. The results suggest the FFQ can be used in the JPHC study cohort I to rank individuals according to intakes for most of the food groups examined. But intake at both population and individual levels was difficult to estimate by the FFQ.
